# Impact of Scoring Single or Multiple Occlusal Lesions on Estimates of Diagnostic Accuracy of the Visual ICDAS-II System

**DOI:** 10.1155/2009/798283

**Published:** 2010-02-09

**Authors:** Anahita Jablonski-Momeni, David N. J. Ricketts, Monika Heinzel-Gutenbrunner, Richard Stoll, Vitus Stachniss, Klaus Pieper

**Affiliations:** ^1^Department of Paediatric and Community Dentistry, Dental School, Philipps University of Marburg, Georg-Voigt-Straße 3, 35033 Marburg, Germany; ^2^Section of Operative Dentistry, Fixed Prosthodontics and Endodontology, Dundee Dental School, University of Dundee, Dundee DD1 4HN, UK; ^3^Department of Operative Dentistry, Dental School, Philipps-University of Marburg, Georg-Voigt-Straße 3, 35033 Marburg, Germany

## Abstract

Carious lesions can occur at different sites on the occlusal surfaces of teeth and may differ in appearance and severity. This study aimed to evaluate how estimates of reproducibility and accuracy of ICDAS-II were affected when all lesions on occlusal surfaces, or only a representative lesion, were scored. 100 permanent teeth with 1–4 investigation sites on the occlusal surface were examined visually by four examiners. Serial sections of the teeth were assessed for lesion depth. Intra- and interexaminer reproducibility (weighted kappa values), sensitivity, and specificity were calculated for all investigation sites and for a randomly selected site per tooth. Comparing the kappa values for the whole sample and the independent sites, no effect or only a small effect was found. Comparing the areas under the ROC-curves no effect could be shown. Examining multiple sites on teeth leads to results comparable to when a single independent site is chosen per tooth.

## 1. Introduction

The turn of the twentieth century has seen the need for improved visual caries detection. In 2001, a systematic review of the literature reported that the strength of the evidence for the diagnostic performance of visual/tactile method for coronal caries was poor due to the small numbers of studies available [1]. For those studies that were included, it was reported that whilst the specificity for the visual/tactile method was high, sensitivity was low.

In the year 2002, an International Consensus Workshop on Caries Clinical Trials (ICW_CCT) was held in Loch Lommond, Scotland and at this meeting a similar systematic review of visual/tactile caries diagnosis was presented [2]. Only twenty nine papers met the inclusion criteria, and the main conclusions were that there was a great variation in the disease process being measured and the examination conditions. In relation to the former conclusion, some studies only recorded cavitated lesions and some noncavitated lesions. Some made an attempt at determining lesion activity, whilst the majority did not. Few gave explicit information about the lesions being recorded and whether they were differentiated from noncarious changes. In relation to the second conclusion, there was no consistency on the use of blunt probes, whether the teeth were cleaned or whether they were dried.

Due to such variation it is clear that comparisons between studies are problematic and the criteria used are not related to the histopathology of the disease. It is these factors that led an International Group to develop the International Caries Detection and Assessment System (ICDAS-II), in the hope that a standardized approach to recording and characterising carious lesions that relate to the histopathology of the disease could be developed for use by researchers, epidemiologists, clinicians, and teachers [3].

Whilst the ICDAS-II can be used on all tooth surfaces, it is noted that carious lesions on the occlusal surface of posterior teeth can occur at different discrete sites and be of different appearance and severity. This is important for the clinician in treating caries, especially when operative intervention is concerned as not all sites may require operative intervention. Clinicians therefore have to make site specific judgements on a single tooth surface. As such, when diagnostic methods or tools are used in laboratory or clinical studies to assess their diagnostic accuracy, the presence of multiple discrete investigation sites on a single occlusal has often been used to increase the sample size. Sites have usually been chosen on the premise that they are discrete and easily relocated to allow assessment of intra- and interexaminer reproducibility [4–6].

However, such studies could and have frequently been criticised at peer review for not having statistically independent data. It can be argued that the position and appearance of a lesion in one part of the fissure system could bias the judgement of the examiner about the appearance of a separate lesion elsewhere in the fissure system and hence skew results.

Bader et al. [7] stated in their systematic review of the performance of methods for identifying carious lesions that in many studies on caries diagnosis the choice of sites rather than surfaces may pose a threat to external validity because most occlusal surfaces will present multiple sites for assessment. The results of site assessment do not summarize the status of the entire surface, which would be important for an epidemiological survey for example. This clearly demonstrates the problem that lesions can vary in severity across the occlusal surface and the dentist's need to evaluate all sites to determine the worse status against a background of differing visual appearances. There are obvious differing needs between the epidemiologist who requires information on the entire surface and the clinician planning operative intervention; both need to be able to determine site specific information against a background of noise.

This study therefore aimed to evaluate how estimates of reproducibility, sensitivity, and specificity of ICDAS-II were affected by whether all lesions on occlusal surfaces, or only a representative lesion, were scored. 

## 2. Materials and Methods

One hundred unrestored permanent molar (*n* = 85) and premolar (*n* = 15) teeth were selected from a group of extracted teeth collected at Dundee and Marburg Dental Schools. These were cleaned thoroughly and stored in water. The teeth were collected and used in accordance with the appropriate legislation and regulations in place in the UK and Germany predating September 2006. 

The teeth used in this study were those used in a previous study [8]. Initially one hundred teeth were selected which had between one to four discrete sites within the pit and fissure system suitable for investigation. In total there were 181 discrete investigation sites which were marked on digital photographs of the occlusal surfaces. Black and white copies, printed in draft quality on plain paper, were used by the examiners during this study and were only suitable for lesion location. The distribution of the investigation sites was: 33 teeth had 1 investigation site, 54 teeth had 2, 12 teeth had 3, and 1 tooth had 4 investigation sites. Only those teeth with two or more discrete investigation sites were used in this study (*n* = 67 teeth) giving a total of 148 investigation sites.

Prior to the visual examination, the reference examiner (D. N. J. R.) trained 3 other examiners (K. P., V. S., and A. J.-M.) in the ICDAS-II classification system in a 2-hour session. For details see Jablonski-Momeni et al. [8]. Each investigation site was visually examined by the four investigators blind to each other using the International Caries Detection and Assessment System (ICDAS-II) (Table 1, http://www.icdas.org/) [9].

After three weeks, 3 of the trained examiners re-examined all of the teeth and investigation sites in order to determine intraexaminer reproducibility. The reference examiner was not available to repeat the examinations, being abroad. 

### 2.1. Histological Preparation

**Figure 1:**
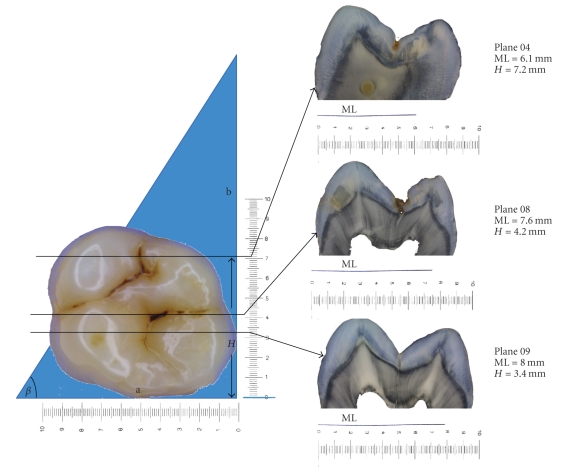
Occlusal view of a molar tooth with three investigation sites and the corresponding histological sections. The length of the embedded coloured foil allows accurate location of the section in the *y*-axis, using the formula *H* = 2 (a - ML). The position of the lesion along each section can be then determined by the *x*-axis coordinate.

Following the visual examination the roots of the teeth were resected using a horizontal cut just apical to the cement-enamel junction as described previously [8, 10]. In brief, a photograph of the occlusal surface of the tooth was taken at the same magnification as a right-angled coordinate system which surrounded the tooth in order that the exact investigation site (IS) could be determined by its *x*- and *y*-coordinates (Figure 1). The mesial surface of each tooth was placed face down in contact with a mounting plate at the base of an embedding cylinder, such that the cut root face was vertical and the distal surface of the tooth was upper most. A right-angled triangle of blue foil with a base to height length of 1: 2 (*β* = 63.5°) was mounted in relation to the cut root surface, parallel with the occlusal surface of the tooth in a vertical direction and with its base aligned horizontally with the mounting plate.

The teeth and foil were embedded in acrylic and sectioned in a bucco-lingual direction, starting from the upper most distal surface. Each cut therefore contained a section of the tooth and the blue foil in the form of a distinct line in relation to the cut root face. If the length of the blue line at the base of the section is ML the formulae determined in Figure 1 (*H* = 2x (a - ML)) will give the height (*H*), or *y*-coordinate of the section. As such the correct section can be determined for each investigation site. As to where this is along each section, the *x*-axis coordinate is measured and marked on an image of the section.

Eleven to fifteen sections were produced per crown (width = 200 *μ*m ± 30 *μ*m) and 1–4 sections per investigation site were available.

### 2.2. Histological Examination

For each investigation site the selected sections were examined by all 4 examiners using a binocular microscope (Wild Heerbrugg AG, Gais, Switzerland) using 16x magnification and reflected light. The *Downer *histological classification system [11] was used to record caries severity at each investigation site and this was carried out blindly to the other examiners (Table 2).

Up to 4 sections were available which could be assigned to each investigation site. A histological score was given to each section and the worst/deepest score was taken as the definitive score for further analysis. Caries extent was based upon colour and structural changes in enamel and dentine, with emphasis being placed on differentiating carious changes from protective changes of the pulp-dentine complex, such as tubular sclerosis and reactionary dentine formation.

For each investigation site the results from all 4 examiners were then compared to achieve a consensus histological score to be used in the subsequent analysis. Where 3 or more examiners agreed on the histological score, this was taken as consensus. Where there was greater disagreement, the sections were reviewed by all examiners and after discussion a consensus was reached.

### 2.3. Data Management and Statistical Evaluation

Both the ICDAS-II and histology scores were recorded on data collection forms and later transferred to an Excel table. For each tooth one investigation site was randomly chosen (SPSS 15.0) in order to avoid dependencies resulting from several measurements on the same tooth. Only those with more than one investigation site were included. Thus the subsequent analyses were carried out for the whole sample (*n* = 148 investigation sites) and the independent sites (independent data *n* = 67).

For the ICDAS-II scores, interexaminer reproducibility was calculated for all pairs of examiners and intraexaminer reproducibility was calculated for the trained examiners using weighted Cohen's kappa (linear weights, ComKappa version 1.0).

The consensus Downer histology was used to calculate sensitivity and specificity at the *D*_1_ and *D*_3_ diagnostic threshold. At the *D*_1_ diagnostic threshold all histological scores 1–4 were classed as caries and each ICDAS-II cut-off was used to calculate sensitivity and specificity for each examiner. Similarly for the *D*_3_ diagnostic threshold histological scores 3 and 4 were classed as caries only and sensitivity and specificity calculated at each ICDAS-II cut-off. Using these sensitivity and specificity values Receiver Operating Characteristic (ROC) analyses were carried out at the *D*_1_ and *D*_3_ thresholds for each examiner.

To test whether kappa values, and the areas under the ROC-curve differed systematically between the two sets of data (whole sample and independent sites), the effect size (Cohen's d) [12] was calculated between the results for all the data and the results for the randomized independent group (SPSS 15.0). According to Cohen [12] when the effect size is between 0.0 to 0.2 it indicates no effect, 0.2–0.5 indicates a small effect, 0.5–0.8 a medium effect, and >0.8 a large effect.

## 3. Results

Initially, 148 investigation sites were planned for statistical evaluation, but, owing to section damage on some teeth only 146 investigation sites and 291 corresponding sections were available for analysis (34 investigations sites with 1 section, 84 with 2 sections, 23 with 3 sections, and 5 with 4 sections).

Three or more examiners agreed on the histological assessment in 82% of investigation sites when using the Downer classification. The interexaminer weighted kappa values were 0.69–0.78 for the histology. Thus the agreement between examiners in the histological assessment of the sections was good to substantial, but where disagreement occurred, a consensus decision was made following discussion and this was used in subsequent analyses.

For examiners 1, 2, 3, and 4 the ICDAS-II scores differed from one site to another in 73.1%, 70.1%, 56.7%, and 77.6% (mean = 69.4%) of teeth, respectively. For the consensus Downer score, in 71.6% of teeth the histological scores also differed from one site to another.

**Figure 2:**
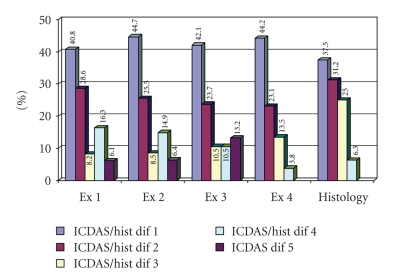
Frequency distribution (%) of the differences between the scores in each surface for each examiner when using ICDAS-II and for the consensus histological scores.

Figure 2 shows the distribution in the differences between the scores at each investigation site in each surface for each examiner when using ICDAS-II and for the consensus histological scores. For example for examiner 1 in 40.8% of the teeth the ICDAS-II score differed from one site to another by an ICDAS-II score of 1, in 28.6% of teeth the score differed by 2, and so forth. It can be seen from the differences in histological score from one site to another that lesions can be of different severity from one site to another on a single tooth and that this is reflected in the differences recorded in the ICDAS-II scoring.

The weighted kappa values for inter- and intraexaminer reproducibility are shown in Table 3. These demonstrate good to substantial agreement between examiners and for each examiner when examinations are repeated. When comparing the kappa values for the whole sample and the independent sites, the effect size was mainly between 0.0 and 0.2 indicating no effect or difference between the two samples.

Table 4 shows for the *D*_1_ and *D*_3_ diagnostic thresholds optimum sensitivity, specificity, the corresponding ICDAS-II cut-off used to achieve this and the area under the curves (AUC) for each examiner when the whole sample and independent sites were analysed. The effect size between the AUCs for the whole data and the randomized independent data shows no effect at the *D*_1_ threshold (effect size between 0.01 and 0.08) or *D*_3_ threshold (effect size between 0.05 and 0.15). 

## 4. Discussion

It has been recognised that detection of caries from a visual examination alone is problematical [1, 2]. The occlusal surface poses a particular problem as this surface is the most commonly affected by caries in children, adolescents, and young adults [13, 14]. The invaginated anatomy and histopathology of the disease process in this surface also complicates early caries detection; initial lesions occur on the walls or at the entrance to the fissure, spread laterally through the enamel and widely and often deeply into dentine before frank cavitation occurs. Subtle changes at the surface of the tooth in previous visual classification systems have not been related to the histopathology of the disease and as such many lesions have been missed or simply not included in the criteria [2]. One of the purposes of the ICDAS-II system is to overcome this short fall, to characterise and describe the earliest visible changes due to caries on all coronal surfaces (and specifically in this study in occlusal surfaces) through to frank cavitation and how these stages relate to the histopathology of the disease.

Even when using a detailed system such as the ICDAS-II there is a degree of subjective interpretation due to perhaps visual perception, lighting, and potential bias. Such bias may arise from other surfaces on the same teeth and in clinical studies of other teeth, or within other areas of the same surface. It might be argued that, for example, if a smooth buccal surface is carious, the likelihood of an approximal surface on the same tooth being carious is much higher. Similarly if the distal fossa of an upper molar tooth is carious it could be argued that the mesial fossa also has a higher risk of being carious and the operator might be inclined to change the visual score to reflect this. However, on the occlusal surface the anatomical sites are often discrete, have individual susceptibilities and risk factors, and it can also be argued that if any technique or detection tool has a place in the dental market they should be able to overcome such biases. One might argue that a blinded design for a study is possible by masking the other sites in the occlusal surfaces with multiple sites so eliminating bias. This could be done on photos (visual diagnosis) or direct on the surfaces. But this would not reflect the in vivo clinical situation when all teeth are subject to visual examination and all sites within one tooth and other teeth would be visible anyway.

Multiple examination sites per occlusal surface have been used in a number of studies. For example, Ferreira Zandoná et al. [15] used premolars with three investigation sites on each occlusal surface to compare the performance of visual examination and laser fluorescence for detection of demineralization in occlusal pits and fissures. They stated that the use of multiple sites on a single tooth provided an opportunity to correlate the methods precisely, and was justified since the sites were well separated and contained distinct demineralization as judged either by histology or by confocal laser microscopy. Lussi et al. [16] used 1–3 sites of 26 occlusal surfaces (total 41 sites) to study the accuracy of an electrical resistance monitor in diagnosing occlusal caries. Subsequent histological examinations revealed no carious connections between multiple test sites on a tooth. The sites were selected according to the following criteria: (1) easily located topographic position, and where there was more than one site on a tooth, the sites were clearly separated. The authors stated that the use of multiple sites in a single tooth was fully justified. It reflects the clinical situation where several occlusal sites on a single tooth require evaluation. In this study no distinction was made between the results whether one site or all of the investigation sites were analysed by the ECM and the potential impact this would have on the apparent diagnostic accuarcy.

This study has clearly shown that the histological severity of caries between investigation sites on the same occlusal surface can vary considerably (Figure 2). However, dentists can also be site specific in making ICDAS-II decisions as the codes given to individual discrete investigation sites on the same tooth surface also differ (Figure 2). The results also show good inter- and intraexaminer reproducibility for the ICDAS-II system and acceptable diagnostic accuracy at both the *D*_1_ and *D*_3_ diagnostic threshold, irrespective of whether the whole sample is analysed or whether one investigation site is randomly chosen to represent independent data for each tooth (effect sizes from 0.00 to 0.25, Tables 3 and 4).

The kappa values for ICDAS-II found in this study are similar to those kappa values published by Ismail et al. [17] who reported that the reliability of six examiners to classify tooth surfaces by their ICDAS carious status ranged between good to excellent (kappa coefficients ranged between 0.59 and 0.91, using two different weighting schemes). In a study where ICDAS-II codes were used in both primary and permanent teeth [18] intra- and interexaminer reproducibility were found to be excellent (kappa values > 0.82). Rodrigues et al. [19] obtained unweighted kappa values of 0.61 for intraexaminer reproducibility (permanent teeth) and 0.51 for interexaminer reproducibility when ICDAS-II was used in permanent teeth.

In many publications it is not always clear how investigation sites were selected. Some authors selected sites which have an easily located topographical position [4, 6]. Or an easily identifiable interlobal groove on the occlusal surface where caries would be likely to occur would be chosen for investigation [5]. In our study we used teeth which had between one to four discrete sites within the pit and fissure system suitable for investigation.

Regarding the Downer histological classification [11], the area under the ROC curve showed good performance of the ICDAS-II in detecting occlusal caries lesions at both *D*_1_ and *D*_3_ diagnostic thresholds (AUC from 0.67 to 0.86, Table 4). Other studies had shown that the ICDAS-II produced areas under the ROC curves of 0.75 [19] and 0.73 [20] which are within the range of our results.

This study supports the view that dentists can be site specific in applying ICDAS-II visual criteria to multiple discrete sites within the same occlusal surface and not necessarily be biased by the appearance at an adjacent site. Dentists' education and clinical experience tell them that lesions can be variable across one tooth surface and the appearance at one site should not dictate the treatment for all sites, each should be taken on their own merit. These results have important implications in relation to the use of human tissues for research, regulations over which, now severely limit the availability and use of extracted teeth for research in many countries.

##  Conflict of Interest

The authors declare that they have no conflict of interest.

## Figures and Tables

**Table 1:** The ICDAS-II criteria.

ICDAS-II code	Criteria [9]
0	Sound tooth surface: no evidence of caries after prolonged air drying (5 seconds)
1	First visual change in enamel: opacity or discoloration (white or brown) is visible at the entrance to the pit or fissure after prolonged air drying, which is not or hardly seen on a wet surface
2	Distinct visual change in enamel: opacity or discoloration distinctly visible at the entrance to the pit and fissure when wet, lesion must still be visible when dry
3	Localized enamel breakdown due to caries with no visible dentine or underlying shadow: opacity or discoloration wider than the natural fissure/fossa when wet and after prolonged air drying
4	Underlying dark shadow from dentine +/− localised enamel breakdown
5	Distinct cavity with visible dentine: visual evidence of demineralisation and dentine exposed
6	Extensive distinct cavity with visible dentine and more than half of the surface involved

**Table 2:** Criteria used in the histological examinations.

Score	Criteria used in the Downer histological examination [11]
0	No enamel demineralisation or a narrow surface zone of opacity (edge phenomenon)
1	Enamel demineralisation limited to the outer 50% of the enamel layer
2	Demineralisation involving the inner 50% of the enamel, up to the enamel-dentine junction
3	Demineralisation involving the outer 50% of the dentine
4	Demineralisation involving the inner 50% of the dentine

**Table 3:** Weighted kappa values for inter- and intraexaminer-reproducibility for visual ICDAS examinations, 95% confidence intervals and the effect size.

	Kappa for all data and 95% CI	Kappa for the randomized data and 95% CI	Effect size
	(*n* = 148)	(*n* = 67)	
*Intraexaminer*-*reproducibility *			
Examiner 2	0.79 (0.71–0.87)	0.76 (0.64–0.88)	0.06
Examiner 3	0.72 (0.64–0.80)	0.72 (0.58–0.86)	0.00
Examiner 4	0.82 (0.74–0.90)	0.82 (0.70–0.94)	0.00
*Interexaminer-reproducibility *			
Examiner 1 versus 2	0.80 (0.72–0.88)	0.79 (0.67–0.91)	0.02
Examiner 1 versus 3	0.68 (0.64–0.72)	0.69 (0.63–0.75)	0.02
Examiner 1 versus 4	0.77 (0.69–0.85)	0.76 (0.64–0.88)	0.00
Examiner 2 versus 3	0.66 (0.62–0.70)	0.60 (0.54–0.66)	0.13
Examiner 2 versus 4	0.75 (0.67–0.83)	0.75 (0.63–0.87)	0.00
Examiner 3 versus 4	0.66 (0.62–0.70)	0.54 (0.48–0.60)	0.25

**Table 4:** The area under the ROC curve, optimum sensitivity and specificity and corresponding ICDAS-II threshold used, for each examiner at *D*_1_ and *D*_3_ diagnostic threshold.

	Examiner 1			Examiner 2			Examiner 3			Examiner 4		

	All data		Randomised	All data		Randomised	All data		Randomised	All data		Randomised
			data			data			data			data
*D*_1_ diagnostic threshold												

Opt Sens	0.70		0.68	0.69		0.66	0.57		0.49	0.71		0.71
Opt Spec	0.83		0.90	0.81		0.80	0.78		0.74	0.73		0.80
ICDAS cut-off		1-2			1-2			1-2			1-2	
AUC (SE)	0.81 (0.04)		0.82 (0.06)	0.80 (0.04)		0.77 (0.07)	0.72 (0.05)		0.67 (0.07)	0.79 (0.04)		0.80 (0.06)
95% CI	0.72–0.89		0.69–0.94	0.72–0.88		0.64–0.89	0.63–0.81		0.53–0.82	0.71–0.87		0.68–0.92
ES for AUC	0.03			0.07			0.08			0.01		

*D*_3_ diagnostic threshold												

Opt Sens	0.71		0.63	0.60		0.54	0.54		0.42	0.75		0.71
Opt Spec	0.88		0.85	0.87		0.83	0.90		0.89	0.80		0.85
ICDAS cut-off		2-3			2-3			2-3			2-3	
AUC (SE)	0.85 (0.03)		0.81 (0.06)	0.84 (0.03)		0.81 (0.05)	0.83 (0.03)		0.78 (0.06)	0.86 (0.03)		0.84 (0.05)
95% CI	0.79–0.92		0.69–0.92	0.78–0.91		0.70–0.92	0.76–0.90		0.66–0.90	0.80–0.92		0.75–0.94
ES for AUC	0.11			0.08			0.15			0.05		

AUC: Area under the curve; SE: Standard error; CI: Confidence interval, ES: Effect size.
